# Globus pallidus internus deep brain stimulation evokes resonant neural activity in Parkinson’s disease

**DOI:** 10.1093/braincomms/fcad025

**Published:** 2023-02-09

**Authors:** Kara A Johnson, Jackson N Cagle, Janine Lobo Lopes, Joshua K Wong, Michael S Okun, Aysegul Gunduz, Aparna Wagle Shukla, Justin D Hilliard, Kelly D Foote, Coralie de Hemptinne

**Affiliations:** Norman Fixel Institute for Neurological Diseases, University of Florida, Gainesville, FL, USA; Department of Neurology, University of Florida, Gainesville, FL, USA; Norman Fixel Institute for Neurological Diseases, University of Florida, Gainesville, FL, USA; Department of Neurology, University of Florida, Gainesville, FL, USA; Norman Fixel Institute for Neurological Diseases, University of Florida, Gainesville, FL, USA; Department of Neurology, University of Florida, Gainesville, FL, USA; Norman Fixel Institute for Neurological Diseases, University of Florida, Gainesville, FL, USA; Department of Neurology, University of Florida, Gainesville, FL, USA; Norman Fixel Institute for Neurological Diseases, University of Florida, Gainesville, FL, USA; Department of Neurology, University of Florida, Gainesville, FL, USA; Norman Fixel Institute for Neurological Diseases, University of Florida, Gainesville, FL, USA; J. Crayton Pruitt Family Department of Biomedical Engineering, University of Florida, Gainesville, FL, USA; Norman Fixel Institute for Neurological Diseases, University of Florida, Gainesville, FL, USA; Department of Neurology, University of Florida, Gainesville, FL, USA; Norman Fixel Institute for Neurological Diseases, University of Florida, Gainesville, FL, USA; Department of Neurosurgery, University of Florida, Gainesville, FL, USA; Norman Fixel Institute for Neurological Diseases, University of Florida, Gainesville, FL, USA; Department of Neurosurgery, University of Florida, Gainesville, FL, USA; Norman Fixel Institute for Neurological Diseases, University of Florida, Gainesville, FL, USA; Department of Neurology, University of Florida, Gainesville, FL, USA

**Keywords:** evoked potentials, local field potentials, globus pallidus, deep brain stimulation, Parkinson’s disease

## Abstract

Globus pallidus internus deep brain stimulation is an established therapy for patients with medication-refractory Parkinson’s disease. Clinical outcomes are highly dependent on applying stimulation to precise locations in the brain. However, robust neurophysiological markers are needed to determine the optimal electrode location and to guide postoperative stimulation parameter selection. In this study, we evaluated evoked resonant neural activity in the pallidum as a potential intraoperative marker to optimize targeting and stimulation parameter selection to improve outcomes of deep brain stimulation for Parkinson’s disease. Intraoperative local field potential recordings were acquired in 22 patients with Parkinson’s disease undergoing globus pallidus internus deep brain stimulation implantation (*N* = 27 hemispheres). A control group of patients undergoing implantation in the subthalamic nucleus (*N* = 4 hemispheres) for Parkinson’s disease or the thalamus for essential tremor (*N* = 9 patients) were included for comparison. High-frequency (135 Hz) stimulation was delivered from each electrode contact sequentially while recording the evoked response from the other contacts. Low-frequency stimulation (10 Hz) was also applied as a comparison. Evoked resonant neural activity features, including amplitude, frequency and localization were measured and analysed for correlation with empirically derived postoperative therapeutic stimulation parameters. Pallidal evoked resonant neural activity elicited by stimulation in the globus pallidus internus or externus was detected in 26 of 27 hemispheres and varied across hemispheres and across stimulating contacts within individual hemispheres. Bursts of high-frequency stimulation elicited evoked resonant neural activity with similar amplitudes (*P* = 0.9) but a higher frequency (*P* = 0.009) and a higher number of peaks (*P* = 0.004) than low-frequency stimulation. We identified a ‘hotspot’ in the postero-dorsal pallidum where stimulation elicited higher evoked resonant neural activity amplitudes (*P* < 0.001). In 69.6% of hemispheres, the contact that elicited the maximum amplitude intraoperatively matched the contact empirically selected for chronic therapeutic stimulation by an expert clinician after 4 months of programming sessions. Pallidal and subthalamic nucleus evoked resonant neural activity were similar except for lower pallidal amplitudes. No evoked resonant neural activity was detected in the essential tremor control group. Given its spatial topography and correlation with postoperative stimulation parameters empirically selected by expert clinicians, pallidal evoked resonant neural activity shows promise as a potential marker to guide intraoperative targeting and to assist the clinician with postoperative stimulation programming. Importantly, evoked resonant neural activity may also have the potential to guide directional and closed-loop deep brain stimulation programming for Parkinson’s disease.

## Teaser

Objective markers are needed to guide deep brain stimulation (DBS) for Parkinson’s disease. Johnson *et al*. established pallidal evoked resonant neural activity as a potential neurophysiological marker to verify the DBS electrode location intraoperatively and identify effective postoperative stimulation parameters.

## Graphical Abstract

**Graphical abstract:**
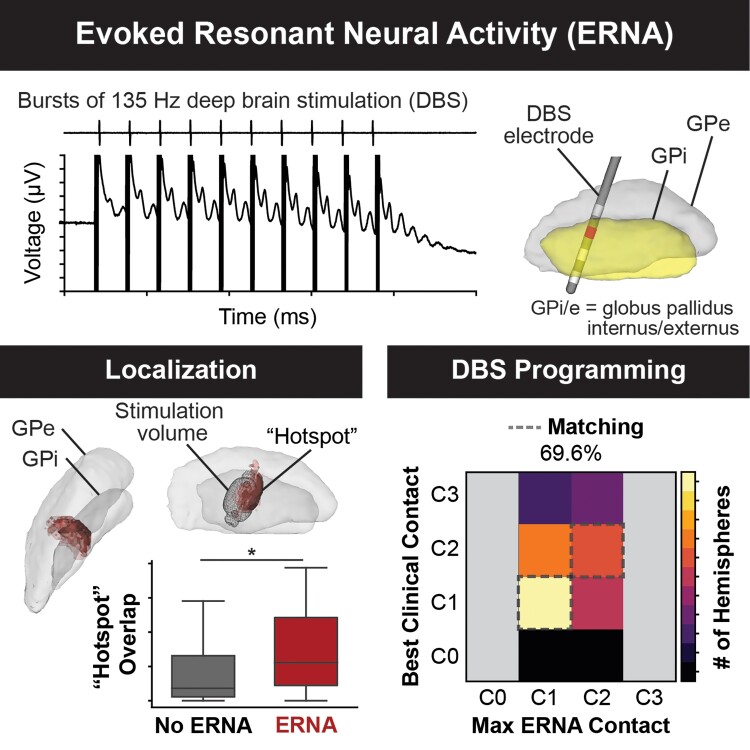

See Steiner and Milosevic (https://doi.org/10.1093/braincomms/fcad033) for a scientific commentary on this article.

## Introduction

Deep brain stimulation (DBS) is a common therapy for medication-refractory Parkinson’s disease that involves surgically implanting electrodes into the basal ganglia and delivering electrical stimulation to modulate neural activity in brain networks and alleviate symptoms. Many individuals who undergo DBS therapy for Parkinson’s disease experience substantial improvement in their Parkinsonian symptoms and quality of life.^1,2^ However, clinical outcomes vary across individuals and several challenges remain that may hinder optimization of the therapy, including determining the optimal lead location and stimulation parameters.

Historically, the subthalamic nucleus (STN) has been the most common brain structure targeted for DBS for Parkinson’s disease. However, the globus pallidus internus (GPi) has been increasingly adopted as a target and has been shown to have advantages over the STN in suppression of dyskinesias, ease of programming, long-term flexibility in medication management, and safety benefits in the setting of mild cognitive decline and/or depression.^3–6^ Clinical outcomes rely on implanting the DBS lead into a precise location within these subcortical structures, and then empirically identifying the optimal stimulation parameters for reduction of symptoms. There has been great interest in investigating markers of therapeutic STN DBS based on imaging^7,8^ or neural signals.^9,10^ Recently, evoked resonant neural activity (ERNA), a high-frequency (∼200–500 Hz) stimulation-evoked response has been proposed as a candidate neural signal. ERNA has been hypothesized to originate from reciprocal connections between the STN and the pallidum, although its exact origins are unclear.^11^ Studies of STN DBS have suggested that ERNA may be localized to the therapeutic target region and may be correlated with postoperative stimulation parameters and possibly reduction in motor symptoms in Parkinson’s disease.^12–15^

Despite robust work on STN ERNA, few studies have focused on identifying markers for GPi DBS or determining the optimal location to stimulate within the GPi target. In this study, we investigated pallidal ERNA as a potential candidate marker to guide DBS therapy for Parkinson’s disease. We aimed to determine if ERNA is a common feature across individuals and if the spatial topography of ERNA could be used to determine specific anatomical substructures. Finally, we aimed to evaluate whether ERNA is correlated with empirically derived postoperative stimulation parameters identified by expert clinicians.

## Materials and methods

### Cohort

Patients undergoing awake GPi DBS implantation surgery for the treatment of Parkinson’s disease at the University of Florida Norman Fixel Institute for Neurological Diseases were included in the study. Patients undergoing DBS implantation surgery targeted to the STN for Parkinson’s disease or the ventralis intermedius (VIM) nucleus for the treatment of essential tremor were included for comparison as controls. Informed consent was obtained for all patients for inclusion in our institutional database (Institutional Review Board #201901807). Both hemispheres were tested in a subset of patients who underwent staged bilateral DBS.

### Surgery

A quadripolar DBS electrode (Medtronic, USA) was implanted with the patient awake according to the surgical team’s standard clinical practice.^4,16,17^ In the main study cohort, the DBS electrode was targeted to the posterolateral GPi (lead model 3387). In the control group, the DBS electrode was targeted to the dorsolateral STN (lead model 3389) for Parkinson’s disease or the VIM thalamic nucleus (lead model 3387) for essential tremor. Each patient underwent multisequence MRI [volumetric gadolinium-enhanced T_1_-weighted sequence, FGATIR (fast grey matter acquisition T_1_ inversion recovery), and FLAIR (fluid-attenuated inversion recovery)] upon which a Schaltenbrand–Bailey atlas was overlaid and deformed to create a patient-specific atlas match.^18^ The MRI scan was then fused to a stereotactic computed tomography (CT) scan with the stereotactic frame (Cosman–Roberts–Wells) in place. A trajectory was planned based on the volumetric imaging and deformed atlas to optimize the lead location within the target structure while avoiding visualized vasculature and the ventricles. Microelectrode recordings and intraoperative macrostimulation (first via the microelectrode sheath, then via the DBS lead to assess thresholds for stimulation-induced side effects and evaluate therapeutic benefit) were used to verify the lead trajectory. The implanted DBS electrode was then connected to an external system for simultaneous recording and stimulation (Neuro Omega, Alpha Omega, Israel).

### Recording and stimulation

**Figure 1:**
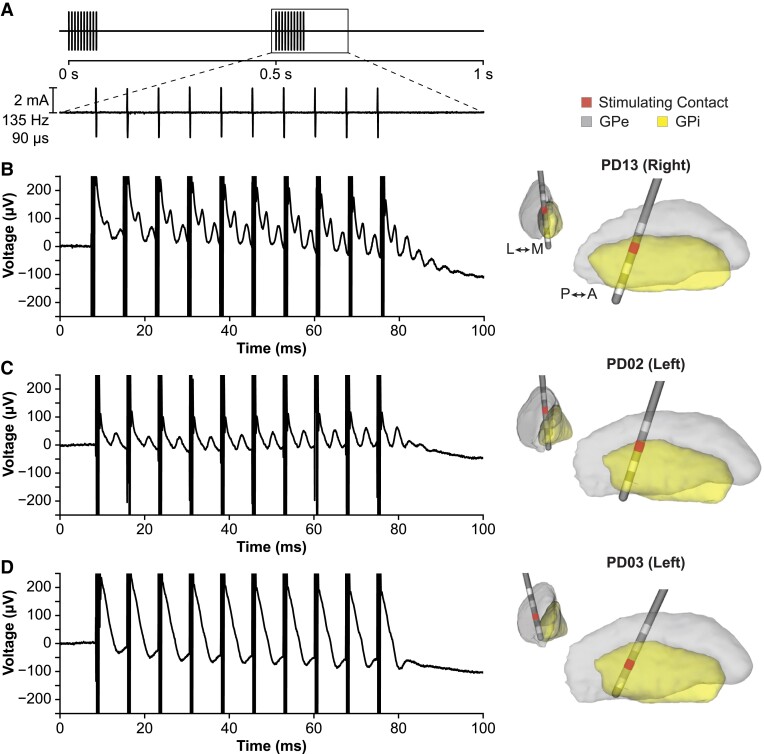
**Examples of evoked responses with GPi DBS.** (**A**) Bursts of high-frequency stimulation were delivered to measure the evoked response. (**B**) Example hemisphere (PD13) showing robust ERNA characterized by high-frequency decaying oscillatory activity between pulses and after the burst of stimulation. (**C**) Example hemisphere (PD02) showing lower amplitude ERNA. (**D**) Only one hemisphere (PD03) did not show the characteristic ERNA when stimulating from any contact. The recordings shown are filtered and averaged across bursts of stimulation; see Supplementary Fig. 1 for corresponding raw recordings from a single burst. Electrode locations and stimulating contacts are shown in the right column.

Local field potentials (LFPs) from all of the DBS contacts were recorded simultaneously at a sampling rate of 22 kHz in monopolar configuration, referenced to a corkscrew electrode (Natus Medical, USA) on the scalp. Similar to previous studies,^12,13^ bursts of stimulation were delivered to elicit ERNA with time in between bursts to measure the evoked response. Monopolar stimulation was delivered from each contact on the DBS lead sequentially from ventral to dorsal. Bursts of 10 pulses of high-frequency stimulation (135 Hz, 2.0 mA, 90 μs, symmetric biphasic waveform) were delivered twice per second for 10 s, for a total of 20 bursts delivered per contact (Fig. 1A). Five seconds of rest (no stimulation) were inserted in between stimulating each contact. As a comparison, 10 s of continuous low-frequency stimulation (10 Hz, 2.0 mA, 90 μs) was delivered from each contact sequentially.

### Signal processing

Signal processing was performed offline with custom Python scripts using similar methods employed in previous studies.^12,13^ The recordings were filtered using a second-order Butterworth high-pass filter (cut-off frequency = 2 Hz). The recordings from the two middle contacts (C1, C2) were bipolar referenced to reduce stimulation artefacts by subtracting the recordings from the two contacts directly adjacent to the stimulating contact (e.g. C0–C2 for stimulation on C1). Due to this bipolar referencing, the subsequent analyses were limited to recordings acquired during stimulation from C1 or C2. Next, the recordings were segmented around the high-frequency bursts and aligned to the last pulse of each burst sequence to analyse the evoked responses across bursts. An 11-point centred moving average filter was applied and a decaying exponential was fit to the recording 4–50 ms after the last pulse and subtracted from each evoked response to remove amplifier baseline trends without altering the decay of the oscillatory peaks. After detrending, the evoked responses were averaged across bursts. For 10 Hz stimulation, the evoked responses were averaged across pulses. A peak-finding algorithm (SciPy, find_peaks: minimum peak prominence = 5 μV, minimum width = 0.5 ms) was applied to the average evoked response to identify ERNA peaks and troughs that occurred ≥4 ms after the pulse to avoid residual stimulation artefacts. ERNA was defined as having at least two peaks and two troughs, and each evoked response was visually checked to verify algorithm accuracy. Manual corrections were required for only 4 (5.0%) of the 80 total evoked responses evaluated (2 stimulating contacts per hemisphere, including all targets). The ERNA amplitude was measured as the peak-to-trough amplitude of the first peak, ERNA frequency was measured as the inverse of the time difference between the first two peaks and the number of peaks detected by the algorithm was counted.

### Electrode localization and computational modelling

Each patient’s preoperative MRI and postoperative CT were transformed to anterior commissure-posterior commissure space and aligned using BRAINSFit rigid registration implemented in the 3D Slicer software.^19^ The DBS electrode location was manually identified using the artefact in the postoperative CT. Each patient’s preoperative MRI was skull-stripped using FreeSurfer^20^ and nonlinearly registered with the PD25 atlas,^21^ a Parkinson’s disease-specific atlas, using the Advanced Normalization Tools (http://stnava.github.io/ANTs/) SyN algorithm.^22^ All right hemisphere DBS electrodes were nonlinearly mapped to the left hemisphere. The resulting transformations were then used to warp all electrode locations and computational modelling results to the PD25 atlas space for direct comparison and visualization with anatomical segmentations.

The volume of tissue activated (VTA) was modelled to estimate the spatial extent of neural activation in response to the intraoperative stimulation. The VTA modelling pipeline has been described in detail previously.^23–26^ Briefly, the voltage solution was computed with a finite element model based on the applied stimulation parameters (monopolar stimulation with C1 or C2, 135 Hz, 2.0 mA, 90 μs) with an encapsulation layer assigned a conductivity of 0.1 S/m, corresponding to a medium impedance state.^27^ The Hessian matrix of second spatial derivatives, or the activating function (AF), was computed to approximate neural activation while accounting for all possible fibre orientations using the AF-3D method detailed in Duffley *et al.*^25^ Established AF thresholds for neural activation^25^ were applied to estimate the VTAs, which were then transformed to each patient’s lead location and warped into the PD25 atlas space for comparison.

### Postoperative stimulation and clinical assessments

Following surgery, all patients underwent postoperative monopolar review and DBS programming optimization by movement disorder neurologists as part of standard clinical care. To determine if ERNA features were correlated with chronic postoperative stimulation parameters, stimulation settings established at the 4-month post-surgery visit were obtained. As is routine at our centre, motor symptoms were assessed by a movement disorder neurologist in the OFF-medication/ON-DBS state using the Unified Parkinson’s Disease Rating Scale (UPDRS) Part III administered at the 4-month visit. The contralateral total motor scores (sum of items 20–26) obtained at the 4-month visit and preoperative baseline (OFF-medication/OFF-DBS) were compared to assess clinical improvement. Contralateral total motor scores were used instead of total motor scores to assess the unilateral effects of stimulation in each hemisphere separately.

### Statistical analysis

All statistical analyses were performed in Python using the SciPy (version 1.7.0) statistical package. Non-parametric tests were used where appropriate: Kruskal–Wallis tests were used for one-way analysis of variance, Mann–Whitney *U* tests or Wilcoxon signed-rank tests were used to compare unpaired or paired distributions, respectively, and Spearman’s rank correlations were used to assess the linear correlation between two variables. For all analyses, the threshold for statistical significance was *P* < 0.05.

### Imaging-based VTA heatmap analysis

Heatmaps were generated to determine whether ERNA varied spatially relative to local neuroanatomical structures and to identify ‘hotspots’ or ‘coldspots’ where stimulation elicited higher or lower ERNA amplitude, respectively. All VTAs were mapped onto a common grid with 0.5 mm isotropic voxels in the PD25 atlas space. First, a heatmap was generated to visualize the spatial distribution of VTAs across the cohort by summing the number of VTAs overlapping at each voxel in the grid (referred to as the *N*-map).

Next, for VTAs that were associated with stimulation that elicited ERNA, the measured ERNA amplitude was assigned to its respective VTA, and voxelwise statistical tests were performed to identify regions that elicited significantly higher or lower ERNA amplitude when stimulated. At each voxel in the grid, a two-tailed unpaired *T*-test was computed between the ERNA amplitudes elicited by the VTAs overlapping at that voxel versus the ERNA amplitudes of the VTAs *not* overlapping at that voxel. The results are presented in the ‘*T*-map’, with corresponding *P*-values reported in the ‘*P*-map’. The resulting *T-*map and *P*-map were thresholded to only include voxels with *N* ≥ 3 overlapping VTAs in the *N*-map.

To correct for multiple comparisons and assess the statistical significance of the heatmap, we implemented permutation testing using methods detailed in Eisenstein *et al*.^28^ Briefly, 1000 permutations were computed in which the ERNA amplitudes were shuffled and randomly assigned to VTAs, and the voxelwise *T-*tests were ran on the shuffled data. The *Q*-statistic was used to assess the overall statistical significance of the *P*-map and was computed for each permutation. Then we calculated the proportion of permutations that resulted in a higher *Q*-statistic than the original map, and the null hypothesis was rejected if this proportion was less than the threshold for significance (*P* < 0.05, corresponding to <50/1000 iterations). Additionally, to identify the ‘hot’ or ‘cold’ spots and to convey relative confidence levels of the statistically significant voxels, we applied clustering thresholds at ≥100 contiguous voxels for *P* < 0.05, *P* < 0.01 and *P* < 0.001. The outlined voxelwise statistical analysis was also repeated for ERNA frequency and the number of peaks.

## Results

A total of *N* = 40 hemispheres (*N* = 35 patients) were included in this study, which comprised 27 GPi (22 patients), 4 STN (4 patients), and 9 VIM (9 patients). The cohort demographics are reported in Table 1 for the patients implanted in the GPi and in Supplementary Table 1 for the patients implanted in the STN or VIM.

**Table 1:** Cohort demographics

Subject	Sex	Age at surgery	Baseline UPDRS-III total score	Target	Hemisphere tested
PD01	M	73	20^a^	GPi	Left
PD02	M	73	34	GPi	Left
PD03	M	50	48	GPi	Left
PD04	F	77	32	GPi	Left, Right
PD05	M	64	42	GPi	Right^b^
PD06	M	65	46	GPi	Left^c^
PD07	M	72	37	GPi	Left
PD08	M	72	20	GPi	Left
PD09	M	50	25	GPi	Left
PD10	M	66	26	GPi	Left
PD11	M	65	31	GPi	Left
PD12	F	79	34	GPi	Left^c^
PD13	F	69	17	GPi	Left, Right
PD14	F	70	20	GPi	Left, Right
PD15	F	70	38	GPi	Right
PD16	M	69	38	GPi	Left, Right
PD17	M	65	31	GPi	Right
PD18	M	74	69	GPi	Right^b^
PD19	M	73	22	GPi	Right
PD20	F	64	41	GPi	Right
PD21	M	61^d^	18	GPi	Left, Right
PD22	M	66	46	GPi	Right
*Group*	6 F/16 M	67.6 ± 7.3^e^	34.0 ± 12.5^e^		15 Left / 12 Right

a Scored ON levodopa medication.

b 135 Hz stimulation tested only.

c 3.0 mA stimulation applied instead of 2.0 mA.

d Age 61 (Right), age 62 (Left).

e Mean ± SD.

### ERNA with pallidal stimulation

In 26 of the 27 hemispheres (21 of 22 patients) tested with pallidal stimulation, a large amplitude evoked response resembling the ERNA previously reported in the STN^12^ was observed, characterized by a large peak (occurring at ∼4–6 ms) followed by successively smaller peaks lasting up to ∼20 ms after the last stimulation pulse. Across all stimulating contacts that elicited ERNA (*N* = 41), we observed ERNA with a median (interquartile range) amplitude of 48.8 (41.2) μV, frequency of 309.9 (124.9) Hz and 3.0 (2.0) peaks. The morphology of ERNA varied among hemispheres, as shown by comparing the two examples shown in Fig. 1B and C. Only one hemisphere did not show ERNA (Fig. 1D).

Similar to previous studies, we also observed ERNA with STN DBS but not with VIM DBS (Supplementary Fig. 2). A comparison suggests that the ERNA amplitude and number of peaks may be lower with GPi DBS versus STN DBS, but GPi and STN DBS elicit similar ERNA frequencies (Supplementary Fig. 3). The ERNA amplitude was not correlated with the amplitude of the stimulation artefact (Spearman correlation, *ρ* = 0.04, *P* = 0.82). Both STN DBS and GPi DBS elicited ERNA that increased in amplitude with each successive stimulation pulse within the burst (Kruskal–Wallis, GPi: *H* = 90.0, *P* < 0.001; STN: *H* = 17.9, *P* = 0.036) (Supplementary Fig. 4), while ERNA amplitude after stimulation remained stable across bursts (GPi: *H* = 10.3, *P* = 0.95; STN: *H* = 3.9, *P* = 1.0).

### Effects of stimulation frequency

**Figure 2:**
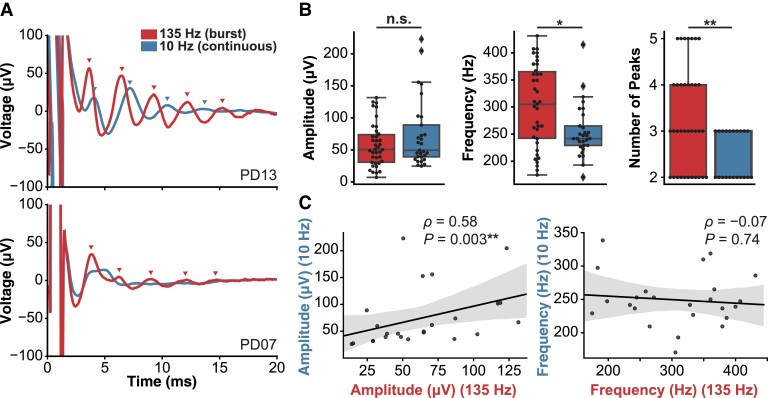
**Effects of DBS frequency on pallidal ERNA.** (**A**) Example hemispheres in which ERNA was elicited by both 135 and 10 Hz stimulation (top panel, PD13) or ERNA was only elicited with 135 Hz but not 10 Hz stimulation (bottom panel, PD07). (**B**) Compared with 10 Hz stimulation, 135 Hz stimulation elicited ERNA with similar amplitude (Wilcoxon signed-rank *W* = 145.0*, P* = 0.90) but with higher frequency (*W* = 60.0, *P* = 0.009) and a higher number of peaks (*W* = 7.0, *P* = 0.004). (**C**) The ERNA amplitudes elicited by 135 and 10 Hz stimulation were significantly correlated across the hemispheres that showed ERNA with both stimulation frequencies (*N* = 14) (left panel) (Spearman *ρ* = 0.58, *P* = 0.003), but the ERNA frequencies were not significantly correlated (right panel) (*ρ* = 0.07, *P* = 0.74).

The evoked responses were compared between high-frequency burst (135 Hz) stimulation and continuous low-frequency (10 Hz) stimulation to determine whether high-frequency stimulation was necessary to reliably elicit ERNA. In the majority of hemispheres tested, ERNA was elicited with both high- and low-frequency stimulation (Fig. 2A, top panel); however, 11/25 hemispheres showed ERNA with only high-frequency stimulation (Fig. 2A, bottom panel). Among the contacts that showed ERNA with both stimulation frequencies (*N* = 24), high-frequency stimulation elicited ERNA with higher frequency (Wilcoxon signed-rank *W* = 60.0, *P* = 0.009) and a higher number of peaks (*W* = 7.0, *P* = 0.004) compared with low-frequency stimulation; however, there was no significant difference between the ERNA amplitudes (*W* = 145.0, *P* = 0.90) (Fig. 2B). Additionally, ERNA amplitudes elicited with high- and low-frequency stimulation were significantly correlated (*ρ* = 0.58, *P* = 0.003), but the ERNA frequencies were not correlated (*ρ* = 0.07, *P* = 0.74) (Fig. 2C).

### ERNA features across stimulating contacts

**Figure 3:**
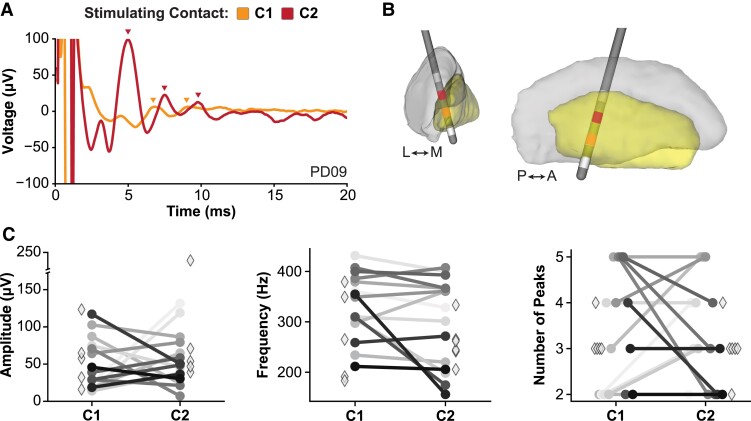
**ERNA characteristics differed across stimulating contacts.** (**A**) Example patient exhibiting higher ERNA amplitude when stimulating from C2 compared with stimulating C1. (**B**) Electrode localization for the example patient in (**A**). (**C**) Comparison of ERNA features across individual hemispheres. ERNA amplitude and the number of peaks showed the greatest differentiation across stimulating contacts, while ERNA frequency was more similar across stimulating contacts. Hemispheres that exhibited ERNA when stimulating from both C1 and C2 are denoted by circles and connected lines, and hemispheres that exhibited ERNA when stimulating from only one contact are denoted by diamonds.

The ERNA amplitude, frequency and number of peaks were compared across stimulating contacts within individuals to determine which metrics may best differentiate between contacts. Of the 26 hemispheres that showed ERNA, 15 hemispheres (57.7%, 13 patients) showed ERNA when stimulating from both C1 and C2 independently, and the remaining 11 hemispheres (42.3%, 11 patients) showed ERNA when stimulating from only 1 contact (C1 or C2) but not the other. As shown in Fig. 3, we observed differences in the elicited ERNA amplitude and number of ERNA peaks across stimulating contacts, but ERNA frequency remained relatively stable within individual hemispheres. This suggests that ERNA may be a localized phenomenon and not the generalized spread of current into the surrounding tissue.

### Anatomical localization of ERNA

The DBS electrode locations and VTAs were mapped to a common atlas space to enable the direct comparison of ERNA localization across patients. The electrodes were clustered in the posterior pallidum spanning both GPi and globus pallidus externus (GPe), but there was spatial variability in the stimulation delivered across the cohort, as shown by the stimulating contact locations and the *N*-map of the VTAs (Supplementary Fig. 5). One patient was excluded from the spatial analysis because accurate electrode localization from the postoperative CT was not possible due to a subdural haematoma.

**Figure 4:**
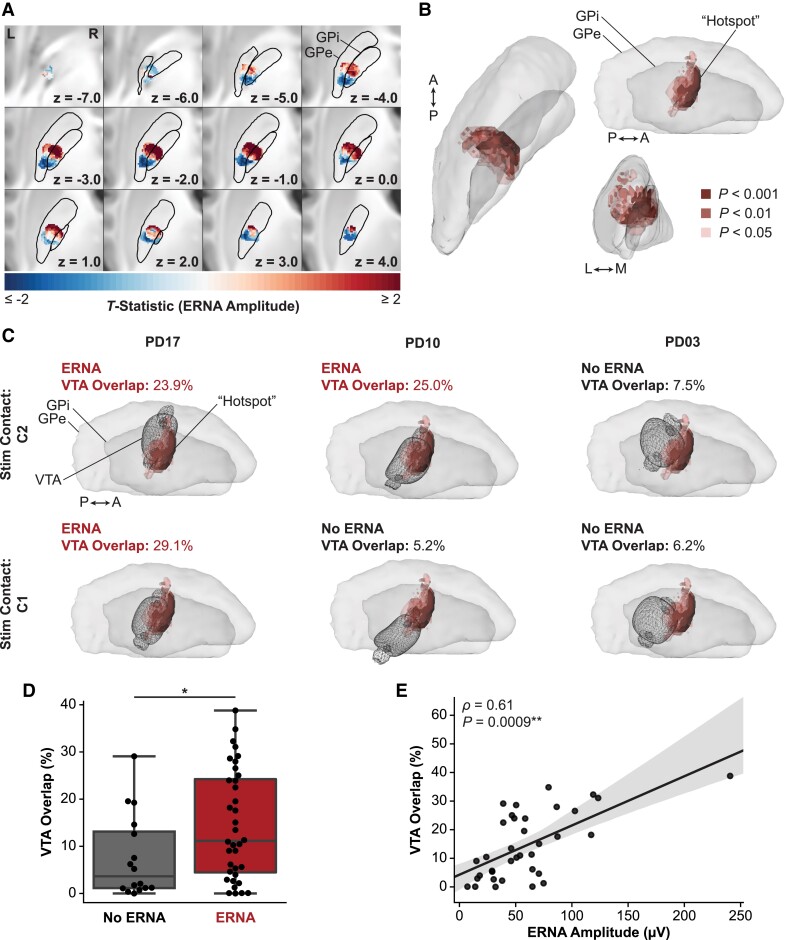
**Heatmap of pallidal ERNA amplitude.** (**A**) Axial slice views of the voxelwise *T*-statistic map, which was validated with permutation testing (using established methods;^28^*P* = 0022). (**B**) The ERNA ‘hotspot’ comprised clusters with ≥100 voxels with *P*-values (derived from voxelwise *T*-tests shown in (**A**)) that met varying statistical significance thresholds. (**C**) Example hemispheres demonstrating that VTAs associated with eliciting ERNA showed higher overlap with the ‘hotspot’ in (**B**) compared with VTAs that did not elicit ERNA. (**D**) At a group level, VTAs that elicited ERNA showed significantly higher overlap than VTAs that did not elicit ERNA (Mann–Whitney *U* = 183.5, *P* = 0.039). (**E**) Among the VTAs that elicited ERNA, the associated ERNA amplitudes were significantly correlated with VTA overlap with the ‘hotspot’ (Spearman *ρ* = 0.61, *P* = 0.009).

Voxelwise *T*-tests were performed to determine whether ERNA amplitude varied spatially relative to the surrounding neuroanatomy and to identify ‘hotspots’ or ‘coldspots’ where stimulation elicited higher or lower ERNA amplitudes, respectively. The resulting *T*-map revealed anterior–posterior and dorsal–ventral gradients in ERNA amplitudes, where relatively anterior–dorsal stimulation elicited higher ERNA amplitudes and relatively posterior–ventral stimulation elicited lower amplitudes (Fig. 4A). Importantly, the *T*-map (and its corresponding *P*-map) was validated using permutation testing (*P* = 0.022).^28^ This *P*-value means that only 22/1000 permutations resulted in a map that was more significant than the true (unpermuted) map.

We then identified a ‘hotspot’ where stimulation elicited higher ERNA amplitudes by further filtering the *T*-map at various thresholds for statistical significance (Fig. 4B). A ‘coldspot’ (*P* < 0.05) was located in relatively posterior and ventral pallidum, but it contained fewer voxels than the cluster size threshold for statistical significance. The ‘hotspot’ that was significantly associated with higher ERNA amplitude (≥100 voxels at *P* < 0.05) was located in the postero-dorsal pallidum spanning both GPi and GPe; however, voxels with the highest statistical significance (*P* < 0.001) were located only within the GPi. Notably, VTAs that elicited ERNA showed higher overlap with the ‘hotspot’ (voxel clusters at *P* < 0.05) than VTAs that did not elicit ERNA, as demonstrated by the examples shown in Fig. 4C and the group-level statistics (*U* = 183.5, *P* = 0.039) (Fig. 4D). Among VTAs that elicited ERNA, overlap of the VTA with the ‘hotspot’ was significantly correlated with the corresponding ERNA amplitude (*ρ* = 0.61, *P* = 0.009), even when excluding one outlier with particularly high ERNA amplitude (*ρ* = 0.57, *P* = 0.003) (Fig. 4E). The voxelwise analyses were also applied to evaluate the spatial relationship of ERNA frequency and the number of peaks, but there were no clear patterns, and the results were not statistically significant.

### Correlation of ERNA with chronic therapeutic stimulation

**Figure 5:**
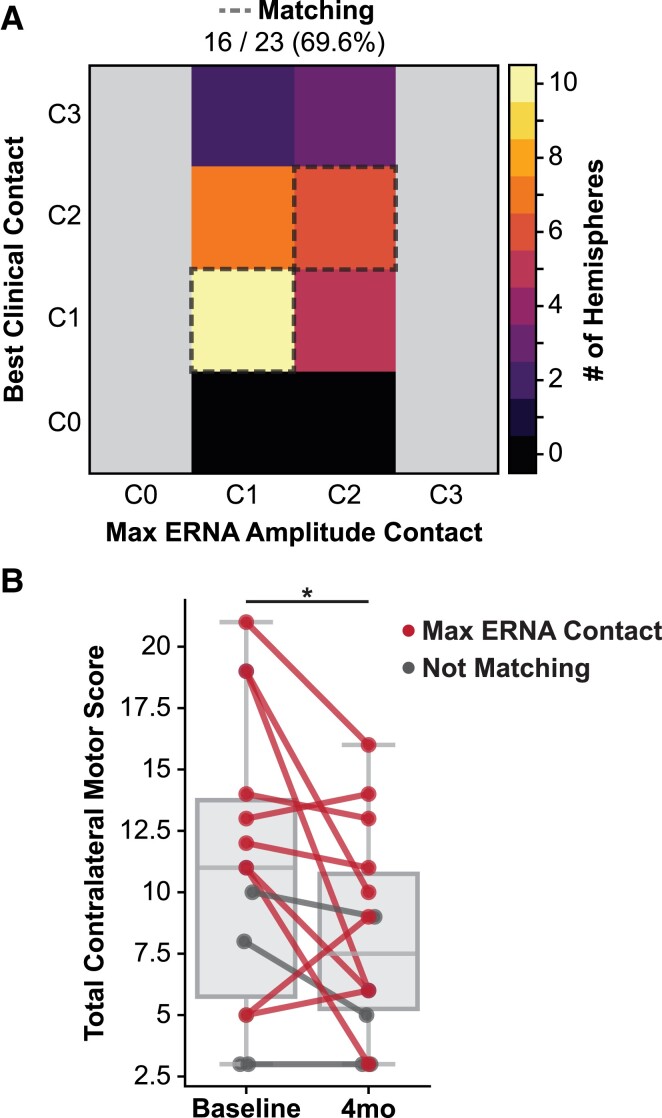
**Correlation of ERNA with postoperative programming and motor improvement scores.** (**A**) Comparison of the number of hemispheres in which the contact that elicited the maximum ERNA amplitude matched one of the contacts selected for postoperative programming at the 4-month clinic visit. (**B**) At the group level, the total contralateral motor scores significantly improved from baseline to 4 months post-surgery (Wilcoxon signed-rank *W* = 13.0, *P* = 0.04). Individual hemisphere scores are shown and coloured based on whether the maximum ERNA amplitude contact matched the postoperative programming contact (*N* = 10) or did not match (*N* = 4).

We evaluated whether ERNA was correlated with therapeutic postoperative stimulation with pallidal DBS. Postoperative stimulation parameters and the stimulating contact that elicited the maximum amplitude ERNA were compared for the hemispheres with stimulation parameters recorded at their 4-month follow-up visit (*N* = 23 hemispheres, 19 patients). In our cohort, 16 of 23 (69.6%) hemispheres were programmed with the contact that also elicited the maximum ERNA amplitude (Fig. 5A). The configurations were considered matching if the maximum ERNA contact was programmed for monopolar stimulation (11/18 hemispheres) or bipolar stimulation (5/5 hemispheres; cathode 1/5, anode 4/5). All of the hemispheres that were considered *not* matching (7/23 hemispheres) were programmed with a contact directly adjacent to the maximum ERNA contact. For example, all three of the hemispheres that were programmed with monopolar stimulation on the most dorsal contact (C3) showed the highest ERNA amplitude when stimulating from C2.

Clinical outcomes were analysed for the patients with UPDRS scores OFF-medication/ON stimulation recorded at their 4-month follow-up visit (*N* = 14 hemispheres; 12 patients). There was a significant reduction in the UPDRS contralateral motor scores compared with preoperative baseline (Wilcoxon *W* = 13.0, *P* = 0.04) with a mean (SD, range) change in scores of −2.9 (4.6, −13.0 to 4.0) points, where a negative change represents a reduction in symptom severity (Fig. 5B). ERNA amplitude was not significantly correlated with baseline UPDRS contralateral motor scores (*ρ* = −0.12, *P* = 0.68). We observed a higher mean improvement in UPDRS contralateral motor scores in hemispheres that were programmed with the maximum ERNA contact (‘matching’) compared with those that were not (‘not matching’) [matching (*N* = 10): −3.6 (5.3, −13.0 to 4.0) points versus not matching (*N* = 4): −1.0 (−3.0 to 0.0) points], but this difference was not statistically significant (*U* = 15.0, *P* = 0.52).

## Discussion

Robust markers associated with therapeutic DBS could play a critical role in guiding DBS for Parkinson’s disease. The precise location of neurophysiological ‘hotspots’ via ERNA or other methodologies has the potential to guide intraoperative targeting, determine effective stimulation parameters for conventional or closed-loop DBS, and improve the efficiency of delivering DBS therapy. The results of this study provide evidence that ERNA is a potential marker that can be practically measured intraoperatively and may yield important information about how to apply DBS most effectively. Although several studies have reported on ERNA with STN DBS, our study is the first to fully characterize ERNA with GPi DBS. Our findings demonstrate that pallidal ERNA was elicited in the majority of hemispheres, and ERNA varied across stimulation frequencies, across individuals and across stimulating locations. Note that although the DBS target was the GPi, we refer to the signal as ‘pallidal ERNA’ since it was elicited by stimulation from contacts in either the GPi or GPe. We discovered a localized ‘hotspot’ where stimulation elicited higher ERNA amplitudes, which indicates that the variability in ERNA was due, in part, to differences in the stimulation location. Critically, the stimulating contact that elicited the highest amplitude ERNA response was blindly selected by expert clinicians for postoperative programming in routine clinical care for a majority of patients. Collectively, our findings suggest that given its spatial topography and correlation with chronic stimulation parameters, ERNA shows promise as an objective marker to guide targeting, stimulation programming and potentially closed-loop algorithms for GPi DBS to treat Parkinson’s disease.

### Characteristics of ERNA

Pallidal ERNA was observed in the majority of hemispheres tested in this study, which suggests that it may be consistent enough to serve as a marker for DBS. Importantly, we replicated previous findings of ERNA in the other commonly applied DBS target, the STN. STN ERNA was observed in all four patients tested, and no ERNA was observed across nine patients with VIM DBS. Our STN and VIM findings were identical to previously published studies.^12,29^ These controls established that our recording and processing techniques were robust and sensitive in detecting ERNA and verified that the measured evoked responses were not artefactual.

All of the previous studies investigating ERNA primarily focused on STN DBS in Parkinson’s disease, and their results collectively suggested that STN ERNA may be useful for targeting or postoperative stimulation parameter selection.^12,14,15^ To the best of our knowledge, only two small studies have investigated pallidal ERNA, both of which report its presence in the majority of the hemispheres tested.^11,29^ Both of these previous studies reported similar ranges in amplitudes and frequencies to those observed in the present study; however, direct comparison should be performed with caution as one study used a paired-pulse stimulation paradigm^29^ and the other studied pallidal ERNA only during STN DBS.^11^ Our preliminary comparison between pallidal ERNA and STN ERNA measured in a small number of patients (*N* = 4) suggested that pallidal ERNA may be lower in amplitude but exhibited a similar frequency and number of peaks. More detailed analyses comparing ERNA with pallidal versus STN DBS are needed.

Additionally, the dynamics of ERNA over time were similar across targets; pallidal ERNA and STN ERNA amplitude increased with successive DBS pulses (Supplementary Fig. 4) but remained relatively stable across bursts of stimulation. However, our protocol was limited to short bursts of stimulation, whereas previous studies show an initial increase in STN ERNA amplitude followed by a decrease over longer stimulation durations.^11,14^ This may be explained by recent studies highlighting the role of synaptic plasticity underlying the suppression of STN activity during high-frequency DBS, potentially through activation of afferent GABAergic projections from the pallidum (specifically GPe).^30^ Performing more detailed comparisons of the temporal dynamics of ERNA in the pallidum and the STN may provide crucial insight into the underlying mechanisms involving synaptic plasticity and dynamics of the pallido-subthalamic circuit. Additionally, these temporal dynamics will be important for determining the feasibility of using ERNA as a control signal for closed-loop DBS paradigms.

### Pallidal ERNA ‘hotspot’

One advantage of our stimulation protocol was that each contact on the DBS electrode was stimulated separately, and therefore, two separate stimulation fields (VTAs), corresponding to high-frequency stimulation delivered from C1 and C2, and the associated evoked responses were tested in each hemisphere. By varying the VTAs within each hemisphere and then mapping all of the VTAs to a common atlas space, we increased our spatial sampling of stimulation in the pallidum to enable a detailed analysis of how stimulation location is related to ERNA. The results show a clear and statistically significant pattern of higher ERNA amplitudes associated with stimulation in the postero-dorsal pallidum, and greater stimulation overlap with this ‘hotspot’ was associated with a higher ERNA amplitude and an increased likelihood of ERNA being elicited.

The heatmap of ERNA amplitudes and the associated ‘hotspot’ suggested that the variability in ERNA amplitude and whether ERNA was elicited could at least partially be explained by where stimulation was delivered. The stimulation location was determined by a combination of the lead trajectory, the location of the stimulating contact(s) (which also depends on the geometry of the lead model) and the stimulation parameters. Therefore, our results suggest that a DBS lead located closer to the ‘hotspot’ may potentially elicit ERNA with a lower stimulation amplitude (i.e. a smaller VTA), whereas a DBS lead located further from the ‘hotspot’ may require a higher stimulation amplitude (i.e. a larger VTA) in order to elicit ERNA. Therefore, subtle differences in the DBS lead location and the applied stimulation parameters will likely directly influence the measured ERNA response, and these differences should be taken into account when interpreting variability. Although this spatial topography adds some complexity to the evaluation of ERNA, it also highlights its potential as a marker for surgical targeting at centres performing intraoperative stimulation testing with the DBS lead. With further clinical validation and spatial analysis, ERNA could potentially be used in conjunction with other sources of data (e.g. microelectrode recordings and side-effect thresholds) to verify that the DBS lead trajectory is satisfactory.

Our heatmap analysis may also provide new insights into the potential mechanisms underlying ERNA in Parkinson’s disease. Simultaneous recordings in the pallidum and STN during STN DBS along with computational modelling suggest that ERNA may arise from modulation of the reciprocal connections between the STN and the GPe.^11,31^ Previous studies in the STN have reported that the highest amplitude ERNA responses were localized to the dorsolateral STN,^12,15^ although detailed atlas-based heatmap analyses of STN ERNA have not been performed. Our results suggest that higher amplitude ERNA may be associated with modulation of specific localized pallido-subthalamic fibres located in the postero-dorsal pallidum. Interestingly, this postero-dorsal ERNA ‘hotspot’ differs somewhat from the reported targeting approaches for pallidotomy^32,33^ and for GPi DBS for Parkinson’s disease.^34^ Given the topographic functional organization of the pallidum and the STN,^35^ the identified ‘hotspot’ projects to downstream basal ganglia-cortical networks, most likely motor and/or premotor networks, that may also be directly or indirectly involved in mediating ERNA. Few studies have investigated the optimal location or networks modulated by GPi DBS that were associated with improvement in Parkinson’s disease. Interestingly, our ERNA ‘hotspot’ coincided with one recent analysis reporting a similar pallidal region associated with higher bradykinesia improvement in Parkinson’s disease that showed a specific connectivity profile to the middle and inferior frontal gyrus.^36^ Future studies should further investigate the cortical and subcortical networks involved in ERNA with GPi DBS versus STN DBS and determine if similar local spatial patterns or networks may be associated with clinical improvement.

### ERNA correlated with postoperative programming

The current DBS standard of care requires hours of programming time per patient for DBS optimization,^37^ and in the era of new directional electrodes and stimulation field shaping, the number of possible stimulation parameter combinations has been exponentially increasing. There is a critical need for data-driven approaches to guide DBS programming. Our results suggest that pallidal ERNA may be a reliable, objective marker that could potentially reduce the complexity of DBS programming. In over two-thirds (69.6%) of hemispheres tested, the contact that elicited the highest amplitude ERNA was chosen for chronic stimulation in routine clinical programming, which was comparable to the proportion (77.5%) recently reported for STN ERNA.^15^ This suggests that pallidal ERNA could improve the efficacy and efficiency of DBS therapy by providing clinicians with information about which contact(s) may be most effective even prior to the patient’s first programming visit.

The patients who were programmed with the maximum ERNA contact also showed a higher average improvement in contralateral motor symptoms when compared with those who were programmed on contacts with lower ERNA amplitude or no ERNA detected. However, this difference was not statistically significant, possibly due to the small subset of patients with complete clinical outcome data available, particularly in the non-matching group. These preliminary results suggest that ERNA measured during intraoperative stimulation may be used to predict contact(s) for postoperative stimulation to provide clinical benefit. Although our cohort included only quadripolar leads, ERNA could be particularly impactful for reducing programming complexity when using newer DBS leads with segmented contacts capable of directional stimulation.^38^ Future studies should further investigate ERNA with segmented DBS leads in order to determine whether it may be useful to establish programming practices that fully leverage emerging directional stimulation capabilities.

The correlation between ERNA and postoperative programming data also indicates that ERNA may be a marker for the stimulation field that produces clinical benefit. All of the hemispheres in our cohort were programmed with either the contact that elicited the maximum ERNA amplitude or a contact directly adjacent. The anatomical location where neural activation occurs in response to DBS is directly related to which contact on the DBS lead is active, in addition to its polarity and the stimulation parameters. Narrowing the contact selection using ERNA could provide an initial estimate of where to stimulate and could improve clinical benefit. Contact location, although perhaps of primary importance, is only one variable that impacts clinical outcomes. Future studies should be directed to whether ERNA could be used to determine effective stimulation amplitudes, pulse widths or frequencies.

### Other candidate markers for GPi DBS

Although the majority of studies have focused on STN DBS, some recent studies have identified neural signals that may show promise to guide GPi DBS for Parkinson’s disease. Similar to the STN, oscillatory power in the beta frequency range (12–30 Hz) is one potential marker that has been explored. Elevated pallidal beta power and beta coherence with the primary motor cortex (M1) have been shown in Parkinson’s disease compared with dystonia, and beta power has been correlated with the severity of akinetic symptoms.^39–41^ Pallidal beta power and pallidal-M1 beta coherence have also been shown to be reduced by therapeutic DBS,^40,42^ which suggests that these neural signals may be a useful marker to determine effective stimulation parameters. However, how oscillatory activity varies spatially within the pallidum remains unclear; this factor would be crucial to determine if beta power (or other frequency bands) could also be used to guide DBS lead placement. Additionally, a systematic comparison of whether beta power could be used to determine contact(s) and stimulation parameters for effective chronic GPi DBS therapy has not been performed. Future studies should further investigate the relationship between beta power and ERNA, as well as compare their predictive power in determining the optimal target and stimulation parameters for GPi DBS in Parkinson’s disease.

### Limitations

Some potential limitations should be considered when interpreting the results of this study. First, we bipolar referenced the LFP recordings to reduce stimulation artefacts and to match the protocols used in previous studies for comparison.^13,14^ As a result, we were limited to analysing the recordings during stimulation from either of the two middle contacts (C1 and C2) due to the ‘sandwich’ referencing scheme. Even with the bipolar referencing, residual stimulation artefacts occurred in some cases; to avoid including these artefacts we only analysed evoked responses ≥4 ms after the stimulation pulse, so short latency evoked responses observed previously^29^ were not included. Based on the surgical targeting approach for GPi DBS used at our centre, the majority of patients in our cohort were programmed on these two middle contacts. However, it is possible that stimulating either the ventral (C0) or dorsal (C3) contacts could elicit higher amplitude ERNA in some hemispheres. In subsequent studies, we will determine if pallidal ERNA can be reliably detected and quantified in a monopolar configuration, which would enable analysis of all stimulating contacts and provide a greater spatial sampling.

The DBS lead locations and VTAs were mapped to a common atlas space to enable direct comparisons across patients using nonlinear image registration. We used established methods that have previously been shown to produce reliable atlas normalization,^43^ and we visually checked that all registration results were reasonable; however, slight differences in the anatomical location of the contacts or VTA in atlas space may occur. Additionally, the VTA model is commonly used to estimate the spatial effects of DBS; however, for this initial study, we used a single VTA model across patients and modelled brain tissue with homogeneous isotropic conductivities. Future studies investigating the spatial pattern of ERNA could incorporate more complex models, such as fibre pathway activation models or patient-specific anisotropic conductivities.^44^

Finally, we compared the contact that elicited the maximum ERNA amplitude with the contact(s) selected for postoperative stimulation and analysed clinical outcomes retrospectively. Notably, we considered contacts programmed with either polarity in our analysis because computational models, including the model used in the present study, have predicted neural activation at both the cathode and anode during bipolar stimulation.^25^ Additionally, intraoperative recordings may not reflect chronic neural signals and may be impacted by a microlesion effect, although this may be less common with GPi DBS than STN.^45^

## Conclusions: ERNA as a marker for GPi DBS in Parkinson’s disease

The present study evaluated pallidal ERNA as a potential marker to guide targeting and postoperative stimulation parameter selection for Parkinson’s disease. Although promising, ERNA has some potential drawbacks. Previous reports have suggested ERNA might be a useful control signal for closed-loop DBS algorithms;^46^ however, it is important to consider that ERNA by itself could not be used as a control signal to turn on stimulation since it is not a spontaneously occurring signal and, therefore, cannot be detected without delivering stimulation. In conjunction with ERNA, another marker (e.g. beta power) would need to serve as the control signal to turn on stimulation. Once stimulation is turned on, it may be possible to adaptively titrate the stimulation based on the ERNA amplitude or frequency, but additional studies are needed to evaluate the temporal dynamics of pallidal ERNA similar to previous studies in the STN^13,14^ and to determine if these metrics would correlate with the symptom improvement in real time. Although our results suggest pallidal ERNA amplitude is not directly correlated with preoperative baseline motor symptom severity, ERNA could potentially be used as a marker of therapeutic stimulation, as evidenced by our correlation with postoperative stimulation parameters. Additionally, due to its high-frequency range (∼200–500 Hz), detecting ERNA will require high sampling rates (≥ 1 kHz) that are not currently available in commercial devices capable of chronic sensing.

Despite these potential limitations in translation as a triggering control signal for closed-loop DBS, ERNA shows promise to guide traditional open-loop GPi DBS in Parkinson’s disease. Our results suggest that pallidal ERNA may be useful to verify satisfactory lead placement intraoperatively or to aid clinicians in postoperative stimulation programming. Prospective validation of these approaches will be required in larger cohorts. Additionally, further studies should investigate whether ERNA could be used to identify effective stimulation parameters to improve symptoms for which the effects of DBS are not immediately observable, such as depression, anxiety or other non-motor symptoms. Identifying objective markers for symptoms that do not typically respond to DBS immediately could help clinicians to reach effective parameters more quickly and thus provide benefits to patients sooner. Future studies should be directed toward investigating the identified ‘hotspot’ associated with higher ERNA amplitudes, including those using directional stimulation and varying stimulation amplitudes within individuals, in order to further uncover the neural structures and networks involved in generating ERNA.

## Supplementary material

Supplementary material is available at *Brain Communications* online.

## Supplementary Material

fcad025_Supplementary_Data

## Data Availability

The data that support the findings of this study are available from the corresponding author upon reasonable request.

## Abbreviations

AF =: activating function
DBS =: deep brain stimulation
ERNA =: evoked resonant neural activity
FGATIR =: fast grey matter acquisition T_1_ inversion recovery
FLAIR =: fluid-attenuated inversion recovery
GPe =: globus pallidus externus
GPi =: globus pallidus internus
LFP =: local field potential
M1 =: primary motor cortex
STN =: subthalamic nucleus
UPDRS =: Unified Parkinson’s Disease Rating Scale
VIM =: ventralis intermedius nucleus
VTA =: volume of tissue activated

## References

[1] Deep-Brain Stimulation for Parkinson’s Disease Study Group, Obeso JA, Olanow CW, et al Deep-brain stimulation of the subthalamic nucleus or the pars interna of the globus pallidus in Parkinson’s disease. N Eng J Med. 2001;345(13):956–963. 10.1056/NEJMoa000827 11575287

[2] Deuschl G , Schade-Brittinger C, Krack P, et al A randomized trial of deep-brain stimulation for Parkinson’s disease. N Eng J Med. 2006;355(9):896–908. 10.1056/NEJMoa060281 16943402

[3] Follett KA , Weaver FM, Stern M, et al Pallidal versus subthalamic deep-brain stimulation for Parkinson’s disease. N Eng J Med. 2010;362(22):2077–2091. 10.1056/NEJMoa0907083 20519680

[4] Okun MS , Fernandez HH, Wu SS, et al Cognition and mood in Parkinson’s disease in subthalamic nucleus versus globus pallidus interna deep brain stimulation: The COMPARE Trial. Ann Neurol. 2009;65(5):586–595. 19288469 10.1002/ana.21596 PMC2692580

[5] Weaver F , Follett K, Hur K, Ippolito D, Stern M. Deep brain stimulation in Parkinson disease: A metaanalysis of patient outcomes. J Neurosurg. 2005;103(6):956–967. 16381181 10.3171/jns.2005.103.6.0956

[6] Wong JK , Cauraugh JH, Ho KWD, et al STN vs. GPi deep brain stimulation for tremor suppression in Parkinson disease: A systematic review and meta-analysis. Parkinsonism Relat Disord. 2019;58:56–62. 30177491 10.1016/j.parkreldis.2018.08.017 PMC8980840

[7] Dembek TA , Roediger J, Horn A, et al Probabilistic sweet spots predict motor outcome for deep brain stimulation in Parkinson disease. Ann Neurol. 2019;86(4):527–538. 31376171 10.1002/ana.25567

[8] Horn A , Reich M, Vorwerk J, et al Connectivity predicts deep brain stimulation outcome in Parkinson disease. Ann Neurol. 2017;82(1):67–78. 28586141 10.1002/ana.24974 PMC5880678

[9] Bronte-Stewart H , Barberini C, Koop MM, Hill BC, Henderson JM, Wingeier B. The STN beta-band profile in Parkinson’s disease is stationary and shows prolonged attenuation after deep brain stimulation. Exp Neurol. 2009;215(1):20–28. 18929561 10.1016/j.expneurol.2008.09.008

[10] Kühn AA , Kempf F, Brücke C, et al High-frequency stimulation of the subthalamic nucleus suppresses oscillatory β activity in patients with Parkinson’s disease in parallel with improvement in motor performance. J Neurosci. 2008;28(24):6165–6173. 18550758 10.1523/JNEUROSCI.0282-08.2008 PMC6670522

[11] Schmidt SL , Brocker DT, Swan BD, Turner DA, Grill WM. Evoked potentials reveal neural circuits engaged by human deep brain stimulation. Brain Stimul. 2020;13(6):1706–1718. 33035726 10.1016/j.brs.2020.09.028 PMC7722102

[12] Sinclair NC , McDermott HJ, Bulluss KJ, et al Subthalamic nucleus deep brain stimulation evokes resonant neural activity. Ann Neurol. 2018;83(5):1027–1031. 29727475 10.1002/ana.25234 PMC6025792

[13] Sinclair NC , McDermott HJ, Fallon JB, et al Deep brain stimulation for Parkinson’s disease modulates high-frequency evoked and spontaneous neural activity. Neurobiol Dis. 2019;130:104522. 10.1016/j.nbd.2019.104522 PMC6879321 31276793

[14] Wiest C , Tinkhauser G, Pogosyan A, et al Local field potential activity dynamics in response to deep brain stimulation of the subthalamic nucleus in Parkinson’s disease. Neurobiol Dis. 2020;143(June):105019. 10.1016/j.nbd.2020.105019 PMC7115855 32681881

[15] Sinclair NC , Mcdermott HJ, Lee W, et al Electrically evoked and spontaneous neural activity in the subthalamic nucleus under general anesthesia. J Neurosurg. 2021;137:449–458. 34891136 10.3171/2021.8.JNS204225

[16] Vitek JL , Bakay RAE, Hashimoto T, et al Microelectrode-guided pallidotomy: Technical approach and its application in medically intractable Parkinson’s disease. J Neurosurg. 1998;88(6):1027–1043. 9609298 10.3171/jns.1998.88.6.1027

[17] Au KLK , Wong JK, Tsuboi T, et al Globus pallidus internus (GPi) deep brain stimulation for Parkinson’s disease: Expert review and commentary. Neurol Ther. 2020;10:7–30. 33140286 10.1007/s40120-020-00220-5 PMC8140010

[18] Sudhyadhom A , Okun MS, Foote KD, Rahman M, Bova FJ. A three-dimensional deformable brain atlas for DBS targeting. I. Methodology for atlas creation and artifact reduction. Open Neuroimag J. 2012;6:92–98. 23091579 10.2174/1874440001206010092 PMC3474940

[19] Fedorov A , Beichel R, Kalphaty-Cramer J, et al 3D slicer as an image computing platform for the quantitative imaging network. Magn Reson Imaging. 2012;30(9):1323–1341. 22770690 10.1016/j.mri.2012.05.001 PMC3466397

[20] Fischl B . Freesurfer. NeuroImage. 2012;62(2):774–781. 22248573 10.1016/j.neuroimage.2012.01.021 PMC3685476

[21] Xiao Y , Lau JC, Anderson T, et al An accurate registration of the BigBrain dataset with the MNI PD25 and ICBM152 atlases. Sci Data. 2019;6(1):210. 31624250 10.1038/s41597-019-0217-0 PMC6797784

[22] Avants BB , Epstein CL, Grossman M, Gee JC. Symmetric diffeomorphic image registration with cross-correlation: Evaluating automated labeling of elderly and neurodegenerative brain. Med Image Anal. 2008;12(1):26–41. 17659998 10.1016/j.media.2007.06.004 PMC2276735

[23] Butson CR , Cooper SE, Henderson JM, McIntyre CC. Patient-specific analysis of the volume of tissue activated during deep brain stimulation. NeuroImage. 2007;34(2):661–670. 17113789 10.1016/j.neuroimage.2006.09.034 PMC1794656

[24] Butson CR , Cooper SE, Henderson JM, Wolgamuth B, McIntyre CC. Probabilistic analysis of activation volumes generated during deep brain stimulation. NeuroImage. 2011;54:2096–2104. 20974269 10.1016/j.neuroimage.2010.10.059 PMC3008334

[25] Duffley G , Anderson DN, Vorwerk J, Dorval AD, Butson CR. Evaluation of methodologies for computing the deep brain stimulation volume of tissue activated. J Neural Eng. 2019;16(6):066024. 10.1088/1741-2552/ab3c95 PMC7187771 31426036

[26] Anderson DN , Osting B, Vorwerk J, Dorval AD, Butson CR. Optimized programming algorithm for cylindrical and directional deep brain stimulation electrodes. J Neural Eng. 2018;15(2):026005. 10.1088/1741-2552/aaa14b 29235446

[27] Butson CR , Maks CB, McIntyre CC. Sources and effects of electrode impedance during deep brain stimulation. Clin Neurophysiol. 2006;117(2):447–454. 16376143 10.1016/j.clinph.2005.10.007 PMC3692979

[28] Eisenstein SA , Koller JM, Black KD, et al Functional anatomy of subthalamic nucleus stimulation in Parkinson disease. Ann Neurol. 2014;76(2):279–295. 24953991 10.1002/ana.24204 PMC4172323

[29] Awad MZ , Vaden RJ, Irwin ZT, et al Subcortical short-term plasticity elicited by deep brain stimulation. Ann Clin Transl Neurol. 2021;8:1010–1023 . 33826240 10.1002/acn3.51275 PMC8108424

[30] Steiner LA , Kühn AA, Geiger JRP, et al Persistent synaptic inhibition of the subthalamic nucleus by high frequency stimulation. Brain Stimul. 2022;15(5):1223–1232. 36058524 10.1016/j.brs.2022.08.020

[31] Hashimoto T , Elder CM, Okun MS, Patrick SK, Vitek JL. Stimulation of the subthalamic nucleus changes the firing pattern of pallidal neurons. J Neurosci. 2003;23(5):1916–1923. 12629196 10.1523/JNEUROSCI.23-05-01916.2003 PMC6741976

[32] Lang AE , Lozano AM, Montgomery E, Duff J, Tasker R, Hutchinson W. Posteroventral medial pallidotomy in advanced Parkinson’s disease. N Eng J Med. 1997;337(15):1036–1043. 10.1056/NEJM199710093371503 9321531

[33] Lozano AM , Lang AE, Galvez-Jimenez N, et al Effect of GPi pallidotomy on motor function in Parkinson’s disease. Lancet. 1995;346(8987):1383–1387. 7475819 10.1016/s0140-6736(95)92404-3

[34] Wong JK , Hilliard JD, Holanda VM, et al Time for a new 3-D image for globus Pallidus internus deep brain stimulation targeting and programming. J Parkinson’s Dis. 2021;11(4):1881–1885. 34420982 10.3233/JPD-212820 PMC8609712

[35] Alexander G , DeLong MR, Strick PL. Parallel organization of functionally segregated circuits linking basal ganglia and cortex. Annu Rev Neurosci. 1986;9(1):357–381. 3085570 10.1146/annurev.ne.09.030186.002041

[36] Sobesky L , Goede L, Odekerken VJJ, et al Subthalamic and pallidal deep brain stimulation: Are we modulating the same network? Brain. 2021;145:251–262. 10.1093/brain/awab258 34453827

[37] Hunka K , Suchowersky O, Wood S, Derwent L, Kiss ZH. Nursing time to program and assess deep brain stimulators in movement disorder patients. J Neurosci Nurs. 2005;37(4):204–210. 16206546 10.1097/01376517-200508000-00006

[38] Schüpbach WMM , Chabardes S, Matthies C, et al Directional leads for deep brain stimulation: Opportunities and challenges. Mov Disord. 2017;32(10):1371–1375. 28843016 10.1002/mds.27096

[39] Silberstein P , Kühn AA, Kupsch A, et al Patterning of globus pallidus local field potentials differs between Parkinson’s disease and dystonia. Brain. 2003;126(12):2597–2608. 12937079 10.1093/brain/awg267

[40] Wang DD , de Hemptinne C, Miocinovic S, et al Pallidal deep-brain stimulation disrupts pallidal beta oscillations and coherence with primary motor cortex in Parkinson’s disease. J Neurosci. 2018;38(19):4556–4568. 29661966 10.1523/JNEUROSCI.0431-18.2018 PMC5943981

[41] Eisinger RS , Cagle JN, Opri E, et al Parkinsonian beta dynamics during rest and movement in the dorsal pallidum and subthalamic nucleus. J Neurosci. 2020;40(14):2859–2867. 32107277 10.1523/JNEUROSCI.2113-19.2020 PMC7117906

[42] Cagle JN , Wong JK, Johnson KA, Foote KD, Okun MS, de Hemptinne C. Suppression and rebound of pallidal beta power: Observation using a chronic sensing DBS device. Front Human Neurosci. 2021;15:1–7. 10.3389/fnhum.2021.749567 PMC8458625 34566612

[43] Ewert S , Horn A, Finkel F, Li N, Kühn AA, Herrington TM. Optimization and comparative evaluation of nonlinear deformation algorithms for atlas-based segmentation of DBS target nuclei. NeuroImage. 2019;184:586–598. 30267856 10.1016/j.neuroimage.2018.09.061 PMC6506227

[44] Chaturvedi A , Butson CR, Lempka SF, Cooper SE, McIntyre CC. Patient-specific models of deep brain stimulation: Influence of field model complexity on neural activation predictions. Brain Stimul. 2010;3(2):65–67. 20607090 10.1016/j.brs.2010.01.003 PMC2895675

[45] Mann JM , Foote KD, Garvan CW, et al Brain penetration effects of microelectrodes and DBS leads in STN or GPi. J Neurol Neurosurg Psychiatry. 2009;80(7):794–797. 19237386 10.1136/jnnp.2008.159558 PMC3791596

[46] Thevathasan W , Sinclair NC, Bulluss KJ, McDermott HJ. Tailoring subthalamic nucleus deep brain stimulation for Parkinson’s disease using evoked resonant neural activity. Front Human Neurosci. 2020;14:71. 10.3389/fnhum.2020.00071 PMC7059818 32180711

