# Supplementary material for: Globus pallidus internus deep brain stimulation evokes resonant neural activity in Parkinson’s disease
Source: Brain Commun. 2023 Feb 9;5(2):fcad025. doi: 10.1093/braincomms/fcad025 (PMC9989134; doi:10.1093/braincomms/fcad025)
Supplement: fcad025_Supplementary_Data [file fcad025_supplementary_data.pdf]

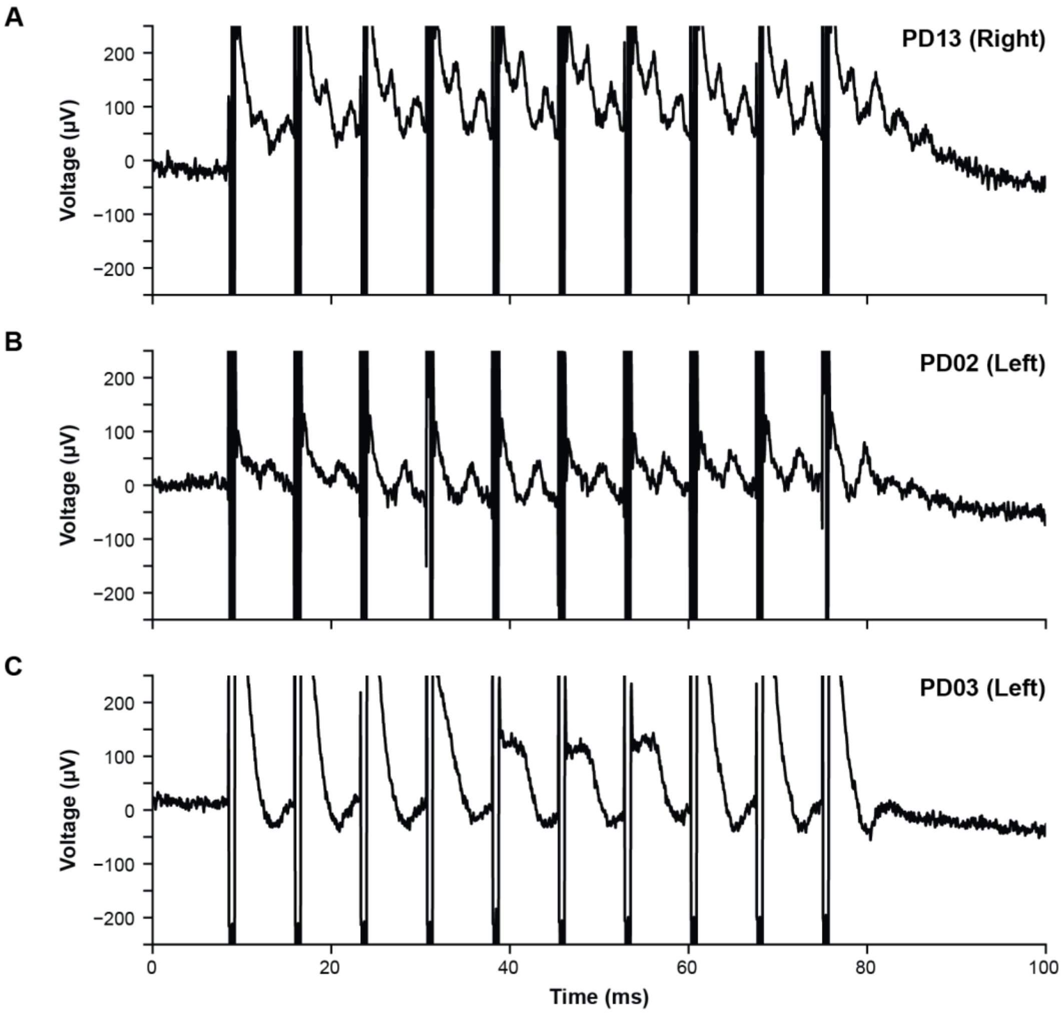

Supplementary Figure 1. Raw recordings of examples of evoked responses with GPi DBS. Panels A-C correspond to the same processed examples presented in Fig. 1B-D.

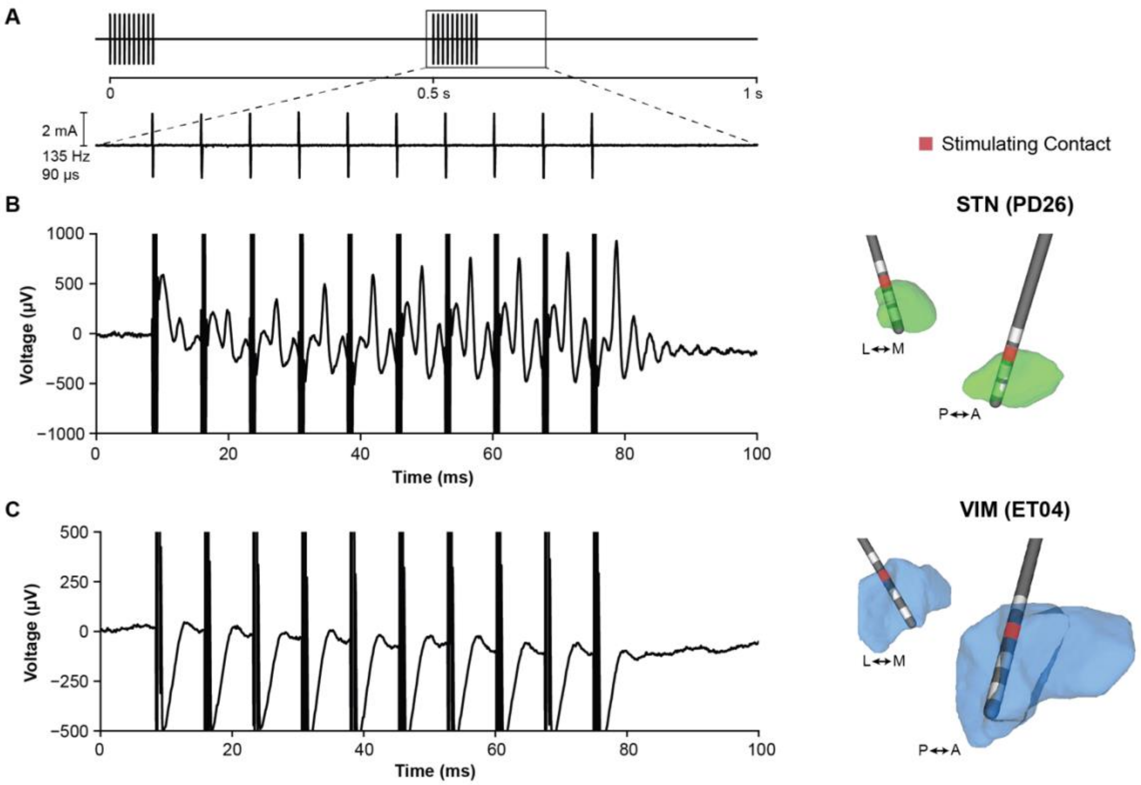

Supplementary Figure 2. Examples of evoked responses with STN DBS and VIM DBS. (A) Bursts of high-frequency stimulation were delivered to measure the evoked response. **(B)** Example STN (PD26) shows characteristic ERNA response similar to those reported previously.[1] **(C)** Example VIM (ET04) showing no ERNA response. Electrode locations and stimulating contacts (highlighted in red) are shown in the right column.

Supplementary Table 1. STN and VIM cohort demographics

Subject	Sex	Age at Surgery	Baseline UPDRS-III Total (PD) or TRS Total (ET) Score	Target	Hemisphere Tested
PD23	M	72	35	STN	Left
PD24	M	71	-	STN	Right
PD25	M	62	27	STN	Left
PD26	M	69	35	STN	Left
**STN Group**	0 F / 4 M	68.5 ± 4.5	32.3 ± 4.6		3 Left / 1 Right
ET01	M	73	33	VIM	Left
ET02	M	82	42	VIM	Right
ET03	F	78	20	VIM	Left
ET04	M	74	44	VIM	Left
ET05	F	79	40	VIM	Left
ET06	M	80	15	VIM	Left
ET07	F	71	27	VIM	Left
ET08	M	79	53	VIM	Right
ET09	F	50	19	VIM	Left
**VIM Group**	4 F / 5 M	74.0 ± 9.7	32.6 ± 13.1		7 Left / 2 Right

- Missing score.

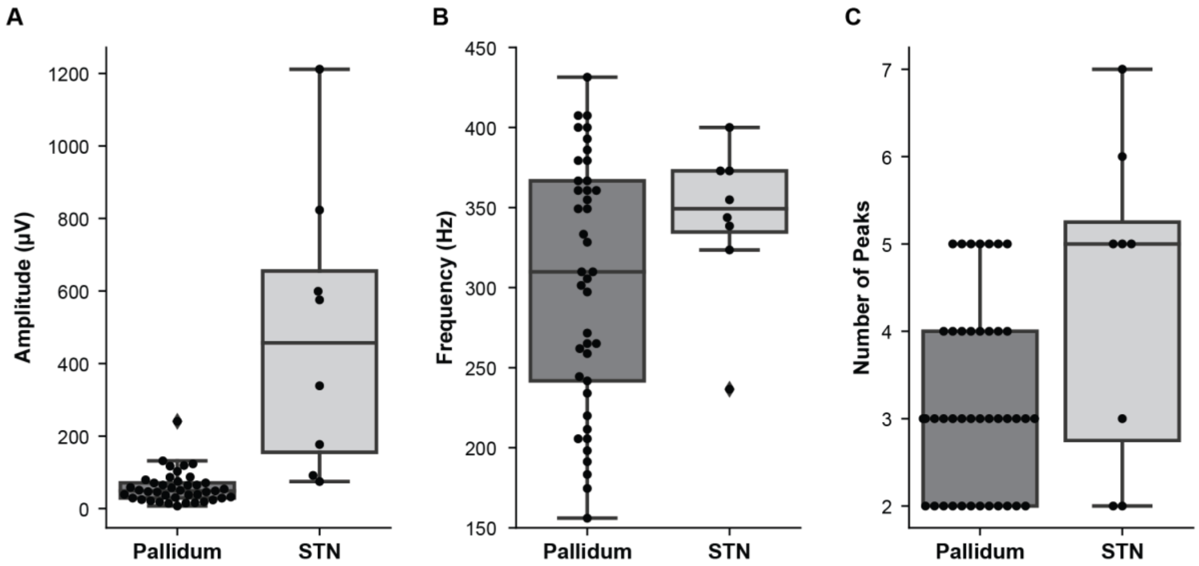

Supplementary Figure 3. Comparison of ERNA metrics with pallidal DBS (*N* = 26 hemispheres) versus STN DBS (*N* = 4 hemispheres) elicited by high-frequency burst stimulation. (A) Pallidal DBS elicited lower amplitude ERNA than STN DBS [median (range): pallidum 48.8 (7.1-240.7) μV versus STN 457.1 (74.7-1211) μV)]. **(B)** Pallidal DBS elicited similar frequency ERNA compared to STN DBS [median (range): pallidum 309.9 (156.0-431.4) Hz versus STN 349.3 (236.6-400) Hz]. **(C)** Pallidal DBS elicited a slightly lower number of ERNA peaks compared to STN DBS [median (range): pallidum 3 (2-5) peaks versus STN 5 (2-7) peaks].

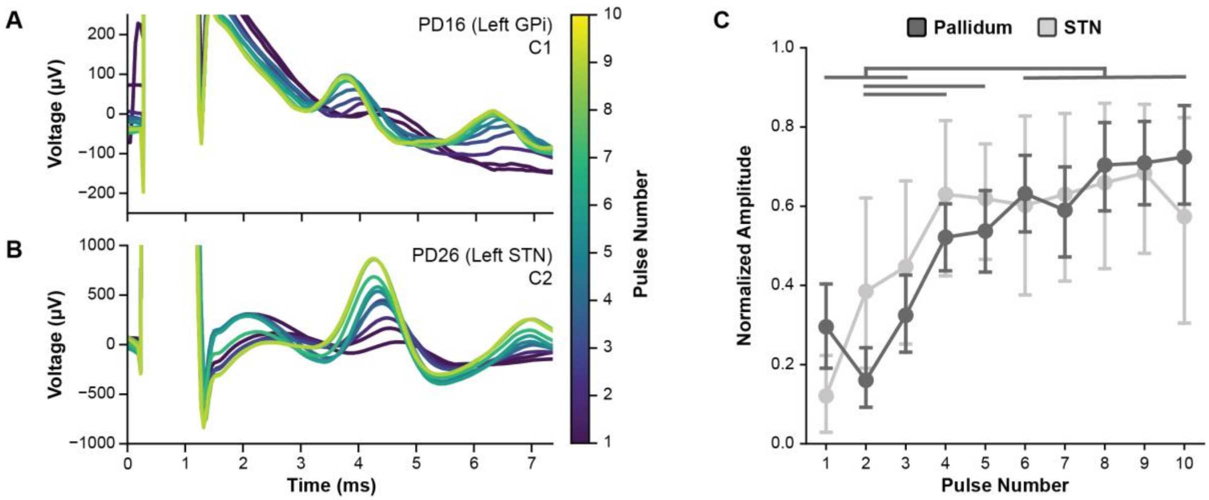

Supplementary Figure 4. Temporal dynamics of ERNA across pulses within bursts of high-frequency DBS. ERNA responses in between pulses shown in **(A)** an example GPi case and **(B)** an example STN case show increasing ERNA amplitude across successive DBS pulses. **(C)** The amplitude of ERNA significantly increased across successive DBS pulses in both targets (Kruskal-Wallis, GPi: H = 90.0, P < 0.001; STN: H = 17.9, P = 0.036). Posthoc testing revealed statistically significant differences across pulses for pallidal DBS using Mann-Whitney U tests with Holm correction for multiple comparisons ($P_{corrected}$ < 0.05 denoted by lines above). For STN DBS, no posthoc comparisons were significant after correction for multiple comparisons (likely due to small sample size).

Methods for Supplementary Figure 4: The bipolar recordings were processed using the same methods outlined in the main text and segmented around the first burst of high-frequency stimulation. The ten pulses within the burst were then temporally aligned for direct comparison. The ERNA amplitude was measured as the LFP voltage at the time of the first peak detected after the burst of stimulation, and then normalized to a range of 0 to 1 for direct comparison across targets. This supplemental analysis was performed on a subset the hemispheres and stimulating contacts in which (1) ERNA was detected after stimulation and (2) the first peak detected after stimulation fell within the interpulse window (7.4 ms for 135 Hz stimulation) (GPi: N = 24 hemispheres / N = 30 contacts, STN: N = 4 hemispheres / N = 8 contacts).

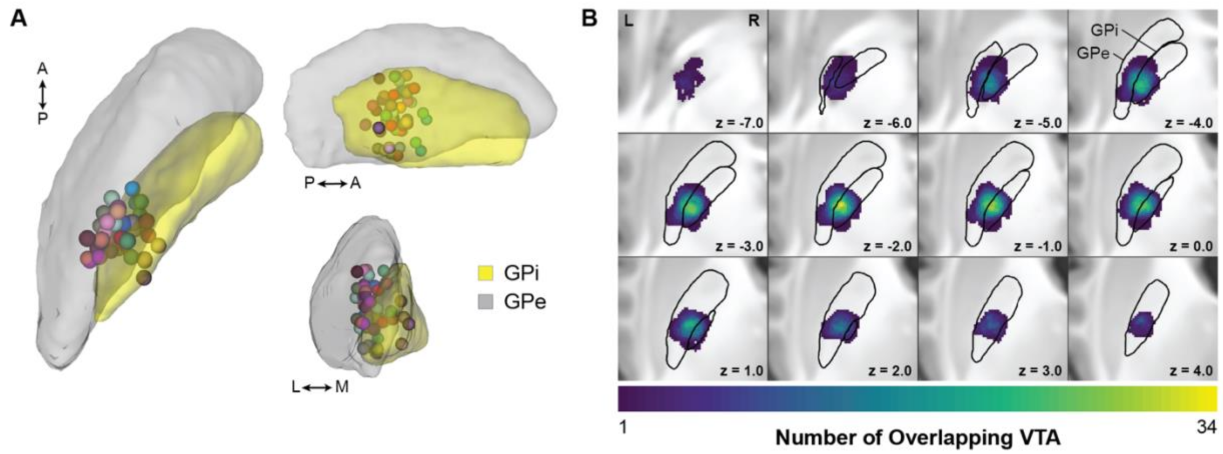

Supplementary Figure 5. Spatial distribution of stimulating contacts and VTAs. (A) Stimulating contacts (C1 and C2) for *N* = 26 subjects in the cohort included in the spatial analysis. One subject was excluded due to inaccurate lead localization. Each sphere represents a contact and are colored by subject. **(B)** Heatmap showing the number of VTAs overlapping at each voxel (*N*-map), which demonstrates the spread of VTAs across the cohort that were used in the voxelwise statistical analyses.

References

1. Sinclair NC, McDermott HJ, Bulluss KJ, et al. Subthalamic nucleus deep brain stimulation evokes resonant neural activity. *Annals of Neurology*. 2018;83(5):1027-1031. doi:10.1002/ana.25234
